# TLR Agonists for Cancer Immunotherapy: Tipping the Balance between the Immune Stimulatory and Inhibitory Effects

**DOI:** 10.3389/fimmu.2014.00083

**Published:** 2014-03-03

**Authors:** Hailing Lu

**Affiliations:** ^1^Tumor Vaccine Group, University of Washington, Seattle, WA, USA

**Keywords:** TLR, cancer immunotherapy, IL-10, Treg, PD-L1

The agonists of toll-like receptors (TLRs) have been actively pursued for their anti-tumor potentials, either as monotherapy or as adjuvants to vaccination or other therapeutic modalities (1). A search on ClinicalTrials.gov using the key words “TLR” and “cancer” returned 34 listings. The idea of using TLR agonist to provide a “danger signal” and break tolerance to tumor antigens has been well embraced by tumor immunologists. However, the promise of TLR agonists-based immunotherapy remains to be realized in the clinic, and only very few TLR agonists have been approved by the FDA. For example, bacillus Calmette–Guerin (BCG) and imiquimod have been approved as standalone therapies, whereas monophosphoryl lipid A (MPL) was approved as a vaccine component. A review of recently published literature on the use of TLR agonists in cancer setting revealed a common mechanism that might have explained the underperformance of TLR agonists as cancer therapeutics: induction of immune suppressive factors that put a break on the TLR agonists-induced inflammation. As shown in Figure 1, TLR agonists have immune stimulatory effects through the induction of costimulatory molecules (CD80, CD86, and CD40) on dendritic cells (DCs) and inflammatory cytokines (TNF-α and IL-12) that polarize Th1 immune response. On the other hand, TLR agonists have immune inhibitory effects as evidenced by the induction of several immune suppressive factors, including IL-10, T regulatory cells (Treg), and PD-L1, all of which could dampen anti-tumor immunity. The following is a brief summary on TLR agonists-induced self-regulatory feedback and the indication for cancer immunotherapy.

**Figure 1:**
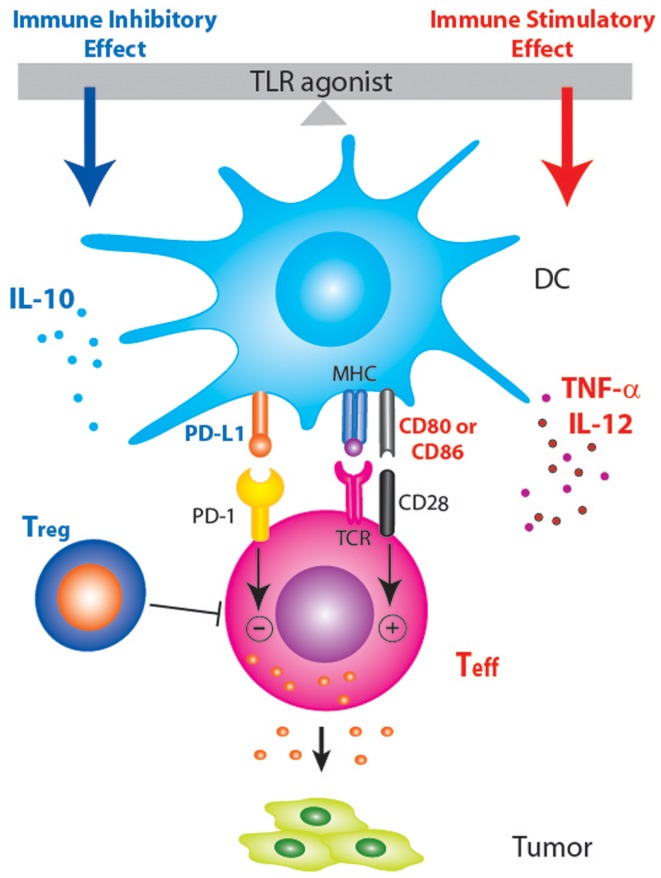
**Schematic diagram showing that PD-1/PD-L1 blockade may enhance TLR-based immunotherapy by tipping the balance between the immune stimulatory and inhibitory effects of TLR agonists**. Treatment with TLR agonists in tumor-bearing host not only induces pro-inflammatory anti-tumor responses but also induce anti-inflammatory factors (IL-10, Treg, and PD-L1) that dampen anti-tumor immune responses.

## Induction of IL-10

IL-10 is an immune suppressive cytokine that inhibits the activity of Th1 cells, thus impeding viral clearance and anti-tumor Th1 immunity. IL-10 could be secreted by different immune cells, including Treg (2), CD4 T cells (3, 4), monocytes, and macrophages (5). The induction of IL-10 by TLR agonists has been demonstrated in infectious disease setting as well as tumor setting. For example, TLR4 signaling with LPS was shown to activate innate IL-10 production in response to *Bordetella pertussis*, which both directly, and by promoting the induction of IL-10-secreting type 1 regulatory T cells (Tr1), inhibit Th1 responses and limit inflammatory pathology in the lungs during infection with *B. pertussis* (6). TLR2 ligation and induction of IL-10 were also shown to suppress immunity against *Candida albicans* (7). Induction of IL-10 also caused the persistence of lymphocytic choriomeningitis virus (LCMV), and IL-10 blockade using a neutralizing antibody can restore T cell immunity and lead to viral clearance (3). IL-10 induction by TLR agonists has been observed in mouse models of breast cancer and melanoma. In MMTV/neu-transgenic mice, a model of human HER2^+^ breast cancer, topical treatment with TLR7 agonist imiquimod induced IL-10, and the major source of IL-10 was Tr1 cells (4). IL-10 induction was also observed in a mouse model of implanted TSA breast cancer, where topical imiquimod was shown to synergize with radiation and low-dose cyclophosphamide in inhibiting tumor growth (8). In a mouse model of B16 melanoma, the induction of IL-10 has also been shown to limit the anti-tumor effects of TLR2 agonist Pam2 lipopeptide (9). Altogether these publications suggest IL-10 induction is probably a common regulatory mechanism that dampens TLR agonists-induced anti-tumor immunity. IL-10 blockade using anti-IL-10 neutralizing mAb significantly enhanced the anti-tumor effects of topical imiquimod (4). The addition of anti-IL-10R to TLR9 agonist CpG also exhibited robust anti-tumor activity exceeding by far that of CpG alone, and elicited anti-tumor immune memory (10). Thus IL-10 blockade holds promise to augment the anti-tumor effects of TLR agonists.

## Induction of Treg

Although some early studies have reported that TLR agonists could inhibit the function or number of Treg (11), more recent studies have demonstrated that TLR agonists can increase Treg number and function. For example, TLR2 ligation has been shown to promote the survival of Treg (12). Treatment of prediabetic mice with a synthetic TLR2 agonist diminished type 1 diabetes and increased the number and function of Treg, also conferring DCs with tolerogenic properties (13). TLR2 agonist Pam3Cys was also shown to induce Treg expansion in the lungs and result in long-term protection against manifestation of allergic asthma in mice (14). Human plasmacytoid DC activated by TLR9 agonist CpG has been shown to induce the generation of Treg (15). Another study reported that TLR agonists-stimulated allogeneic pDCs induces CD8^+^ Tregs that inhibit allogeneic T cell responses, including memory T cells (16). Studies from our group demonstrated that TLR7 agonist imiquimod induces Tregs, either as monotherapy or as an adjuvant to vaccination (4, 17). Treg was induced in both the periphery and the tumor microenvironment (4). Another study showed that imiquimod enhanced the suppressor function of Treg cells by sensitizing Treg cells to IL-2-induced activation (18). A study on transcutaneous vaccination using imiquimod as adjuvant showed that Treg and IL-10 act independently to counter-regulate the cytotoxic T lymphocytes (CTL) response induced by vaccination (19). When TLR9 agonist CpG was used as adjuvant to protein vaccination, antigen-specific Treg was induced (20). The ability of CpG to induce Treg has been shown to be mediated by p38 MAPK and inhibition of p38 in DC was shown to attenuate Treg induction by TLR agonists and enhance their efficacy as vaccine adjuvants and cancer immunotherapeutics (21). Thus inhibiting Treg induction represents another opportunity to augment the anti-tumor effects of TLR agonists.

## Induction of PD-L1

PD-L1 (also known as B7-H1 or CD274) is a B7-related protein that inhibits T cell activation via engaging the programed death-1 (PD-1) receptor that is expressed on activated T cells. PD-L1 can be expressed on tumor cells as well as hematopoietic cells. The induction of PD-L1 by TLR ligation has been reported for different TLR agonists, in both *in vitro* and *in vivo* studies. For example, TLR7/8 agonist resiquimod and TLR4 agonist LPS have been shown to induce PD-L1 on DC and contribute to the development of tolerogenic APCs (22). TLR4 ligation by MPL was also shown to enforce the tolerogenic properties of oral mucosal Langerhans cells (23). Topical imiquimod was shown to induce PD-L1 as well as CD86 in different subsets of skin DC (24). Another study showed that TLR4 agonist LPS and TLR7/8 agonist CL097 induced PD-L1 expression on macrophages and the induction appeared to be dependent on IL-10 (25). Induction of PD-L1 has been linked to the lack of protective immunity to bacteria (26). In mouse tumor models, the induction of PD-L1 has also been shown to be an important mechanism that limits the anti-tumor efficacy of TLR agonists (27, 28). The TLR3 agonist poly I:C up-regulated PD-L1 on DC, and depletion or blockade of PD-L1 on activated DCs increased the magnitude of effector CD8 T cell expansion (28). PD-L1 also collaborates with Treg to impair the recall responses of tumor-specific memory T cells (27). Combination of PD-L1 blockade, CD4 T cell depletion, and tumor cell vaccination resulted in complete regression of large established RENCA tumors and established long-lasting protective immunity (27). Thus, blocking PD-1/PD-L1 signaling represents another opportunity to augment the anti-tumor effects of TLR agonists.

It should be noted that the list above is not exhaustive. Other immune suppressive factors such as TGF-beta (TGF-β), mostly notably secreted from Treg, could also be induced after TLR agonist treatment, especially with agonists of TLR2 and TLR4 (13, 29). It should also be noted that the various immune suppressive factors listed above don’t function separately but in an interactive manner. For example, induction of PD-L1 is crucial to the induction of Treg (30), and the induction of PD-L1 has been shown to be dependent on IL-10 (25). TLR ligation could induce these different suppressive factors simultaneously, and blocking one factor may decrease the other factors as well.

In summary, the immune responses are highly controlled. Once a T cell response is initiated, it needs to be dampened to prevent collateral damage. TLR ligation not only initiates immune response, but also triggers negative regulatory pathways. The above discussion about TLRs is equally true for the normal existent immune response to cancer as well as cancer vaccine-induced response. The induction of these regulatory pathways represents a major obstacle in developing TLR agonists as cancer immunotherapeutics, and the promise of using TLR agonists to eradicate tumor cells probably won’t be realized unless we block the negative regulators (IL-10, Treg, and PD-L1, etc) and tip the balance toward an overwhelming pro-inflammatory response. Cancer immunologists are now exploring novel combinational therapies that combine TLR ligation with blockade of the negative regulators. For example, the cancer immunotherapy trial network (CITN) has considered a pilot clinical trial testing the combination of topical imiquimod and IL-10 blockade in breast cancer patients with chest wall metastasis (per communication with Dr. Martin Cheever, CITN director). Unfortunately this idea did not move forward due to the unavailability of anti-human IL-10 mAb. Currently a clinical trial combining topical imiquimod and PD-1/PD-L1 blockade for treating breast cancer cutaneous metastasis is also being planned in the University of Washington.

The early data from clinical trials of anti-PD-1 and anti-PD-L1 are harbingers of a radical change in immunotherapy as well as cancer therapy in general. Based in large part on data from the check-point inhibitor trials, it’s been predicted that cancer immunotherapy will provide the backbone of up to 60% of cancer therapy within the next decade. There are at least seven companies competing in the anti-PD-1/PD-L1 space. The agents will be tested with every conceivable combination. Combinations with immunotherapies that augment existent immune responses or vaccine-induced immune responses, such as TLR therapies will be tested soon with anti-PD-1/PD-L1 and are highly likely to be more effective in that context. Not only will the blockade of PD-L1 signaling likely enhance the immune stimulatory effects of TLR agonists, but we also expect TLR ligation to enhance the effect of PD-1/PD-L1 blockade by increasing tumor infiltrating immune cells (TIL). The presence of TIL has been associated with good clinical response in the check-point blockade therapy. In addition to anti-PD-1/PD-L1, there will be other check-point inhibitors that will also become available for testing with TLR agonists. The world of cancer immunotherapy is on a predictable course to become a major component of cancer therapy and TLR agonists will likely play an important role.
